# Preferential Neuronal Responses to Snakes in the Monkey Medial Prefrontal Cortex Support an Evolutionary Origin for Ophidiophobia

**DOI:** 10.3389/fnbeh.2021.653250

**Published:** 2021-03-24

**Authors:** Ha Trong Dinh, Hiroshi Nishimaru, Quan Van Le, Jumpei Matsumoto, Tsuyoshi Setogawa, Rafael S. Maior, Carlos Tomaz, Taketoshi Ono, Hisao Nishijo

**Affiliations:** ^1^System Emotional Science, Faculty of Medicine, University of Toyama, Toyama, Japan; ^2^Department of Physiology, Vietnam Military Medical University, Hanoi, Vietnam; ^3^Research Center for Idling Brain Science (RCIBS), University of Toyama, Toyama, Japan; ^4^Primate Center and Laboratory of Neurosciences and Behavior, Department of Physiological Sciences, Institute of Biology, University of Brasilia, Brasilia, Brazil; ^5^Laboratory of Neuroscience and Behavior, CEUMA University, São Luis, Brazil

**Keywords:** snakes, phobia, rostral anterior cingulate cortex, amygdala, snake detection theory

## Abstract

Ophidiophobia (snake phobia) is one of the most common specific phobias. It has been proposed that specific phobia may have an evolutionary origin, and that attentional bias to specific items may promote the onset of phobia. Noninvasive imaging studies of patients with specific phobia reported that the medial prefrontal cortex (mPFC), especially the rostral part of the anterior cingulate cortex (rACC), and amygdala are activated during the presentation of phobogenic stimuli. We propose that the mPFC-amygdala circuit may be involved in the pathogenesis of phobia. The mPFC receives inputs from the phylogenically old subcortical visual pathway including the superior colliculus, pulvinar, and amygdala, while mPFC neurons are highly sensitive to snakes that are the first modern predator of primates, and discriminate snakes with striking postures from those with non-striking postures. Furthermore, the mPFC has been implicated in the attentional allocation and promotes amygdala-dependent aversive conditioning. These findings suggest that the rACC focuses attention on snakes, and promotes aversive conditioning to snakes, which may lead to anxiety and ophidiophobia.

## Introduction

Specific phobia is a type of anxiety disorder with a lifetime prevalence ranging from 3% to 15% across various countries (Eaton et al., 2018). Patients with specific phobia display immediate excessive fear or anxiety in response to a specific object or situation despite the absence of actual danger imposed by the specific object or situation, and the disturbances by specific phobia impose a significant impact on patients’ daily activity (American Psychiatric Association, 2013). Specific phobia consists of five types, and ophidiophobia (snake phobia) belongs to an animal type of specific phobia. Psychological studies reported that 53.3% of survey participants felt anxiety in response to snakes (Davey, 1994), and 2–3% of the participants were ophidiophobia-like (Klorman et al., 1974; Klieger, 1987; Polák et al., 2016), and that ophidiophobia is one of the most common specific phobias (Fredrikson et al., 1996).

It has been proposed that this specific phobia may have an evolutionary origin (Mineka and Öhman, 2002; Russell et al., 2015; Rakison, 2018). The fear module of primates, including the superior colliculus, pulvinar, and amygdala, may have evolved particularly in response to the threat posed by snakes (Isbell, 1994, 2006, 2009; Öhman and Mineka, 2003; Soares et al., 2017). Consistently, monkey pulvinar neuronal activity is more sensitive to snake images than other control images (Le et al., 2013, 2014, 2016). Furthermore, human and monkey behavioral studies support the existence of an innate fear module sensitive to snake images. Human infants (5–6 months old) who likely had less experience of snakes looked at snake images longer than control images and displayed stronger sympathetic responses to snake images (Hoehl et al., 2017; Rakison, 2018), while young children and adults detected snakes faster than control images (LoBue and DeLoache, 2008; Masataka et al., 2010). In monkeys, isolation-reared (snake-naïve) mouse lemurs and pig-tailed macaques avoided a snake model and odor of snakes (Nelson et al., 2003; Weiss et al., 2015). Also, humans are predisposed to efficiently learn aversive conditioning between snakes and unconditioned shock (Seligman, 1970; Öhman and Mineka, 2003). These findings suggest that the innate fear module for snake detection and learning may be involved in snake phobia.

It has been proposed that attentional bias to specific animals may promote anxiety (i.e., phobia; Matthews and MacLeod, 2002; Heeren et al., 2013; LoBue and Rakison, 2013): images of specific animals are automatically processed in a fear module regardless of attention (Öhman and Soares, 1994), compete with cognitive activity, and when the activity for the specific animals exceeds cognitive activity, capture attention for awareness to induce anxiety. Consistent with this hypothesis, snake images, which are prone to induce phobia (see above), captured more attention than other control images in monkeys and humans (Shibasaki and Kawai, 2009; Soares et al., 2014; Kawai and Koda, 2016; Gomes et al., 2017). It is suggested that the anterior cingulate cortex (ACC) is involved in attentional allocation to emotional stimuli (Niedenthal and Kitayama, 1994) and that the affective (rostral) part of the ACC (rACC) is involved in salience evaluation of emotional stimuli (Bush et al., 2000). Furthermore, phobogenic stimuli were detected faster than control stimuli in subjects with strong fear comparable to specific phobia and patients with phobia (Öhman et al., 2001; Bar-Haim et al., 2007), while the rACC was activated in response to phobogenic stimuli including snakes in patients with specific phobia (Rauch et al., 1995; Pissiota et al., 2003; Carlsson et al., 2004; Amir et al., 2005; Straube et al., 2007; Britton et al., 2009). Thus, the currently available data suggest that the ACC, especially rACC, plays an important role in the pathogenesis of ophidiophobia.

Based on evolutional theories for the origin of phobias (see above), we hypothesized that the ACC, which receives projections from the pulvinar (Porrino et al., 1981; Romanski et al., 1997), is also sensitive to snakes. Consistently, neurons in the medial prefrontal cortex (mPFC) including the ACC responded faster and stronger to snakes in monkeys (Dinh et al., 2018). Furthermore, behavioral studies reported that monkeys, as well as humans, are more sensitive to striking postures in snake detection and threat assessment (Masataka et al., 2010; Etting et al., 2014). Based on these behavioral findings and evolutionary reasoning, here we tested whether mPFC neurons are more responsive to images of snakes with striking postures in non-human primates.

## The Behavioral Task for Neurophysiological Recording from The Monkey mPFC

To test this prediction, we recorded and analyzed monkey mPFC neuronal responses to snake images with striking and non-striking postures. The recording was performed using two adult macaque monkeys (*Macaca fuscata*: 1 female and 1 male) weighing 7.1–8.6 kg, which was the same monkeys used in the previous experiment (Dinh et al., 2018). In the previous study (Dinh et al., 2018), the responses of mPFC neurons to snake images regardless of their postures were compared with other categories of images (e.g., raptors, carnivores, humans, and monkey faces, et cetera). In this study, the neuronal responses to snake images with striking and non-striking postures were analyzed. While the monkeys were individually housed with food available *ad libitum*, the monkeys’ water intake was restricted in their home cages and they obtained juice rewards during training as well as recording experiments (Dinh et al., 2018). The monkeys were treated according to the United States Public Health Service Policy on Human Care and Use of Laboratory Animals, the National Institutes of Health Guide for the Care and Use of Laboratory Animals, and the Guidelines for the Care and Use of Animals at the University of Toyama. Experimental procedures in this study have been approved by the Ethical Committee for Animal Experiments at the University of Toyama (Dinh et al., 2018).

**Figure 1:**
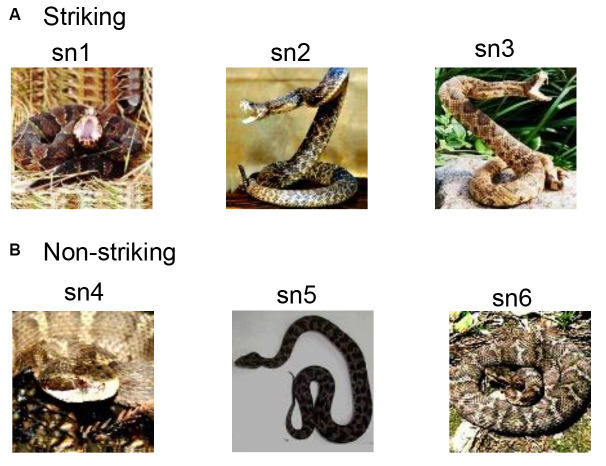
Visual stimuli used in a neurophysiological recording from the monkey medial prefrontal cortex (mPFC). **(A)** Three photos of snakes with striking postures. **(B)** Three photos of snakes with non-striking postures. The same visual stimuli used in the study by Le et al. (2014) were also used in the present study.

The detailed procedures are described in Dinh et al. (2018). Briefly, the monkey sat in a monkey chair in front of a computer display. Eye-movements were monitored by a CCD camera (Matsuda, 1996). In the present study, six snake photos consisting of three snake images with striking postures and another three snake images with non-striking postures were used (Figure 1). The present study used the same visual stimuli in the previous study in the monkey pulvinar (Le et al., 2014). The monkeys discriminated against the snake images in a sequential delayed nonmatching-to-sample task (DNMS; Dinh et al., 2018). Briefly, the task started with a buzzer tone, followed by a fixation cross for 1.5 s. Then, a sample stimulus was presented for 500 ms (sample phase) between 1 and 4 times with a 1.5-s interval. After a 1.5-s interval from the last presentation of the sample stimulus, a new stimulus (target phase) was presented. The monkey could obtain a juice reward if the monkey pressed a button in the chair within 2 s.

Upon reaching a 96% correct-response criterion in the DNMS task, a U-shaped epoxy-resin plate was attached to the skull under anesthesia (Nishijo et al., 1988; Tazumi et al., 2010; Dinh et al., 2018). The monkeys were retrained with the DNMS task two weeks after the surgery, while the head was painlessly fixed using a head-restraining device for the U-shaped plate. After re-training of the monkeys with the head fixed, neuronal activity was recorded from the mPFC during the DNMS task. The detailed procedures for electrophysiological recording were reported previously (Dinh et al., 2018). Briefly, a glass-coated tungsten microelectrode was stereotaxically inserted into the mPFC to record neuronal activity. All the data, including analog signals of neuronal activities, were stored in a computer off-line analysis.

## Characteristics of mPFC Neuronal Responses to Snake Postures

**Figure 2:**
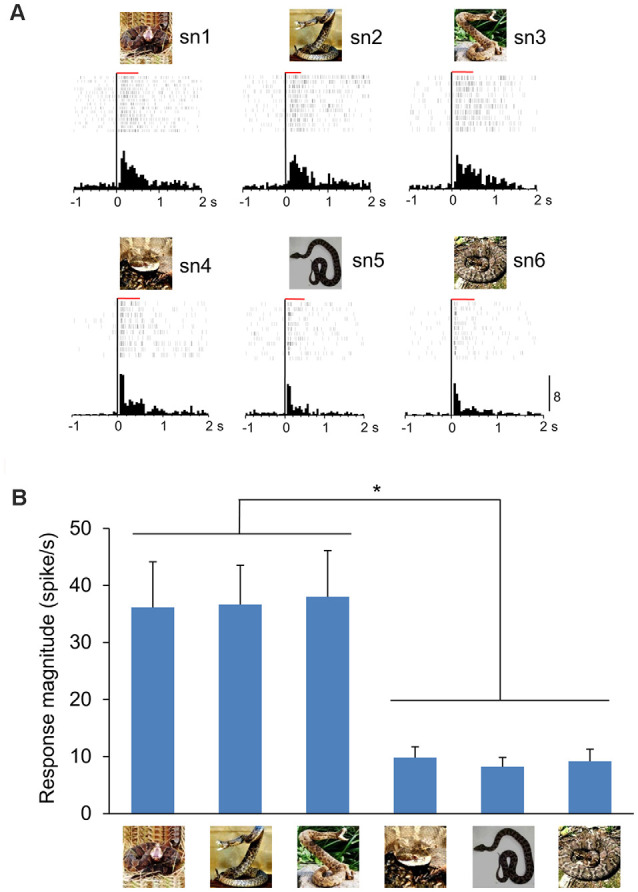
A representative mPFC neuron sensitive to snake postures. **(A)** Peri-event histograms of the mPFC neuronal activity in response to each snake photo. Each raster display above each histogram indicates neuronal activity. The red horizontal bars above the raster display indicate the stimulus presentation period (500 ms). Zero in the abscissas indicates the stimulus onset. Calibration at the right bottom of the figure indicates the number of spikes per trial in each bin. Bin width = 50 ms. **(B)** Response magnitudes of this neuron to the six snake images. Histograms indicate mean ± SEM. *p < 0.05.

Only neuronal responses to sample stimuli were analyzed (Dinh et al., 2018). Briefly, significant responses to each snake image were determined by a Wilcoxon signed–rank test (*p* < 0.05), in which neuronal activity during 500-ms after (post) stimulus onset was compared with that during 100-ms before (pre) stimulus onset. The mPFC neurons were defined as snake-responsive neurons if a given mPFC neuron showed significant responses to at least one of the six snake images. The 235 mPFC neurons were tested with all six snake images in the DNMS task, and 95 neurons responded to one or more snake images (snake-responsive neurons). The numbers of mPFC neurons and snake-responsive neurons in the individual monkeys are indicated in Supplementary Table 1. There were no significant differences in the proportion of snake-responsive neurons among the mPFC neurons between the two monkeys (*χ*^2^-test; $\chi_{\left(1\right)}^{2}$ = 1.147, *p* = 0.284). Figure 2 shows a representative snake-responsive mPFC neuron. The neuron responded stronger to snakes with striking postures (Figure 2A). For each snake-responsive neuron, response magnitudes to each stimulus were computed [i.e., (mean firing rates during the post 500 ms period) minus (mean firing rates in the pre 100 ms period)]. There was a significant difference in the response magnitudes among the six snake images in this neuron (one-way ANOVA: *F*_(5,63)_ = 6.215, *p* < 0.0001; Figure 2B). Subsequent *post hoc* tests indicated that response magnitudes to the three snake images with striking postures were larger than those to the three snake images with non-striking postures (Tukey test, *p* < 0.05).

**Figure 3:**
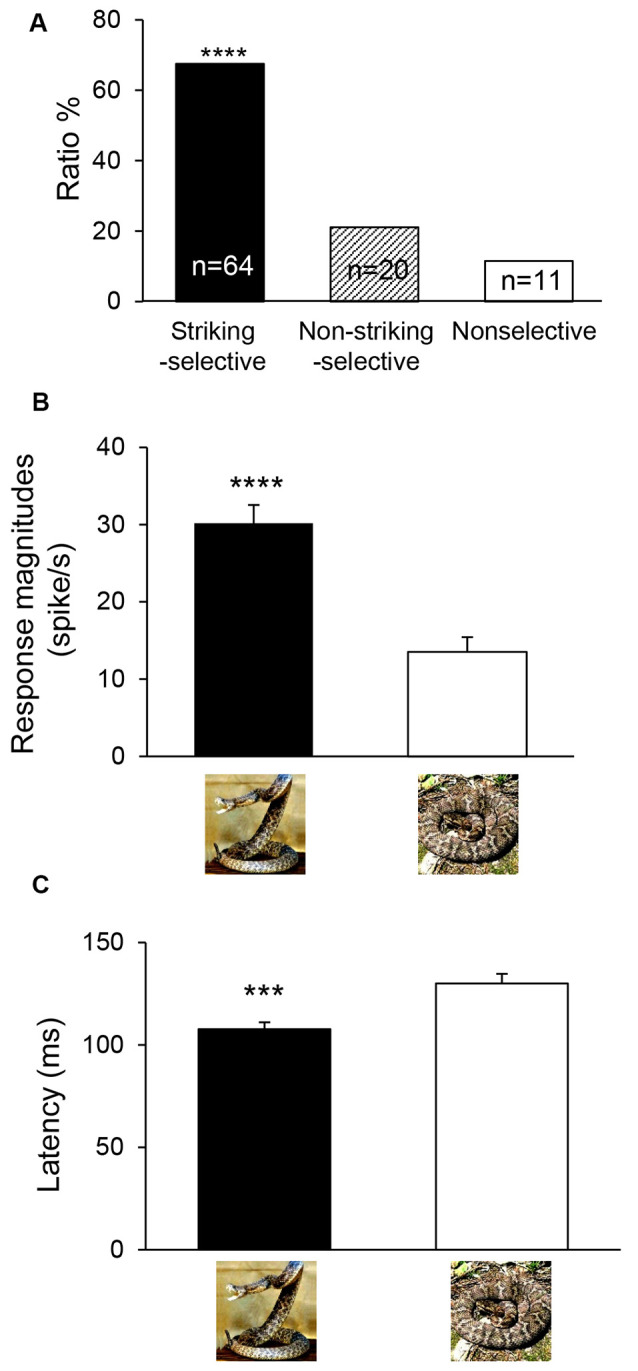
Classification **(A)** and response characteristics **(B,C)** of snake-responsive neurons. **(A)** The ratios of three types of mPFC neurons to the snake postures. ****Significant difference from non-striking-selective mPFC neurons (*p* < 0.0001). **(B)** Comparison of mean response magnitudes to snakes with striking postures and those to non-striking postures (*n* = 95). ****Significant difference from snakes with non-striking postures (*p* < 0.0001). **(C)** Comparison of mean response latencies to snakes with striking postures and those to non-striking postures (*n* = 87). ***Significant difference from snakes with non-striking postures (*p* < 0.001).

Snake-responsive neurons were further categorized based on comparison of response magnitudes to the snake images with striking postures with those to non-striking postures. For this categorization, two peri-event histograms, one for the snakes with striking postures and the other for the snakes with non-striking postures, were constructed. The mPFC neurons were categorized as striking-selective neurons if the response magnitudes to the three snake images with striking postures were larger than those to the three snake images with non-striking postures (unpaired *t*-test, *p* < 0.05), while the mPFC neurons were categorized as non-striking-selective neurons if the response magnitudes to the three snake images with non-striking postures were larger than those to the three snake images with striking postures (unpaired *t-test*, *p* < 0.05). The remaining mPFC neurons were defined as nonselective neurons. Of the 95 snake-responsive mPFC neurons, 64 neurons responded stronger to snakes with striking postures (striking-selective), 20 neurons responded stronger to snakes with non-striking postures (non-striking-selective), and 11 neurons showed no difference in response magnitudes between these two categories of the snake images (non-selective; Figure 3A). Since there were no significant differences in the proportion of striking-selective neurons among the mPFC neurons between the two monkeys (*χ*^2^-test; $\chi_{\left(1\right)}^{2}$ = 0.095, *p* = 0.758), the combined data from the two monkeys were further analyzed. The ratio of the striking-selective neurons was significantly greater than that of the non-striking-selective neurons (*χ*^2^-test; $\chi_{\left(1\right)}^{2}$ = 20.51, *p* < 0.0001). Furthermore, averaged response magnitudes of all snake-responsive neurons to the snakes with striking postures and those to the snakes with non-striking postures were compared by paired *t*-tests (*p* < 0.05). The results indicated that the averaged response magnitudes to the snakes with striking postures were significantly larger than those to the snakes with non-striking postures (paired *t*-test: *t*_(94)_ = 5.459, *p* < 0.0001; Figure 3B).

Response latencies to the snake images were also analyzed. For each neuron, the same two data sets used to analyze response magnitudes for snakes with striking and non-striking postures (see above) were also used to analyze response latencies. Response latency of the mPFC neuron was measured as the period between the stimulus onset and the time point at which the neuronal firing rate exceeded the mean ± 2 SD of the baseline firing rate. The mean response latencies of all striking-selective neurons were compared to non-striking-selective neurons by paired *t*-tests (*p* < 0.05; *n* = 87 for latency measures). The results indicated that averaged response latencies to the snakes with striking postures were significantly shorter than those to the snakes with non-striking postures (paired *t*-test: *t*_(86)_ = 3.804, *p* = 0.0003; Figure 3C).

## Population Coding of Snake Postures in The Monkey mPFC

To investigate temporal changes in population coding of snake postures in the mPFC, the initial 200-ms after stimulus onset was divided into four 50-ms epochs: epoch 1 (0–50 ms), epoch 2 (50–100 ms), epoch 3 (100–150 ms) and epoch 4 (150–200 ms). The mean response magnitude in each epoch was similarly calculated, as follows: (mean firing rate in each epoch) minus (mean firing rate during the 100-ms pre-period). These four data sets were separately analyzed by multidimensional scaling (MDS). MDS can convert the relationships (Euclidean distances as dissimilarity in this study) between all possible pairs of stimuli to a geometric representation of the stimuli in a space (see Young and Hamer, 1987 for more details). In this study, data matrices of response magnitudes in a 95 × 6 array (95 snake-responsive neurons × 6 stimuli) were generated in each epoch and were subjected to MDS analysis. Then, six visual stimuli were positioned in the two-dimensional space so that the geometrical relationships among the stimuli represented the original relationships (MDS, PROXSCAL procedure, SPSS statistical package, version 16; Shepard, 1962; Kruskal, 1964).

**Figure 4:**
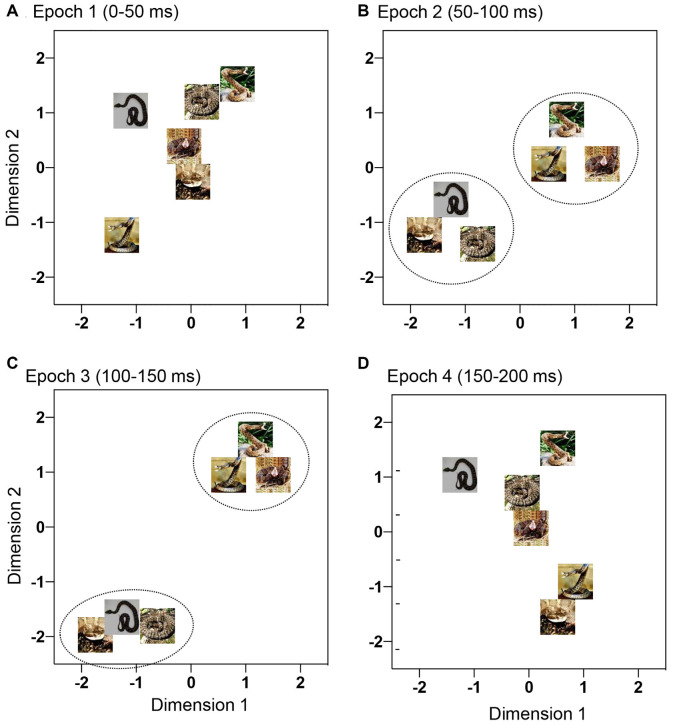
Distributions of the six snake photos in a 2-D space derived from multidimensional scaling (MDS) analysis of response magnitudes of the 95 mPFC neurons to these photos. **(A–D)** Response magnitudes were analyzed by MDS epoch 1 **(A)**, epoch 2 **(B)**, epoch 3 **(C)**, and epoch 4 **(D)**. In epochs 2 and 3 **(B,C)**, the snakes with striking postures were separated from the snakes with non-striking postures (*p* < 0.05).

In the 2-D MDS spaces, *r*^2^ values of epochs 1, 2, 3 and 4 were 0.671, 0.819, 0.953, and 0.762, respectively. The distributions of the six snake images in 2D space derived from each epoch data are shown in Figure 4. In epoch 2 and 3, two clusters, one for snakes with striking postures and another for snakes with non-striking postures, were recognized. Discriminant analyses of the MDS data indicated that the correct percent of discrimination between the two categories of the snake images was 97.32% and 100% in epochs 2 and 3, respectively (*p* = 0.016 and *p* = 0.012, respectively; Table 1). In epochs 1 and 4, the two categories of the snake images were not significantly separated.

**Table 1:** Discriminant analyses of the multidimensional scaling (MDS) results in the medial prefrontal cortex (mPFC).

**Epochs**	**Correct ratio (%)**	*p*
**Epoch 1 (0–50 ms)**		
Striking vs. non-striking	56.3	0.127
**Epoch 2 (50–100 ms)**		
Striking vs. non-striking	100	0.016
**Epoch 3 (100–150 ms)**		
Striking vs. non-striking	100	0.012
**Epoch 4 (150–200 ms)**		
Striking vs. non-striking	65.2	0.271

A previous study reported that response magnitudes and latencies in the mPFC were positively correlated with the corresponding data in the pulvinar (Dinh et al., 2018). Furthermore, in the previous study in which responses to the same snake images with striking and non-striking postures were analyzed in the pulvinar, snakes with striking postures were significantly separated from those with non-striking postures in epoch 2 in the MDS analysis (Le et al., 2014). This study investigated how the MDS data for the snakes with striking and non-striking postures in the mPFC (this study) were related to those in the pulvinar (Le et al., 2014). To investigate the relationships between the two MDS results, we analyzed the relationships between the Euclidean distances between all stimulus pairs in MDS spaces in epochs 2 and 3 in the mPFC and those in the pulvinar data in epoch 2 (Le et al., 2014) by linear regression analysis. The results indicated that the distances between all possible stimulus pairs in the mPFC MDS in epoch 2 were significantly and positively correlated with the corresponding data in the pulvinar MDS space in epoch 2 (*r^2^* = 0.717; *F*_(1,13)_ = 32.97, *p* < 0.0001), and that the distances in the mPFC MDS in epoch 3 were also significantly and positively correlated with the corresponding data in the pulvinar MDS space in epoch 2 (*r^2^* = 0.754; *F*_(1,13)_ = 39.74, *p* < 0.0001). These findings suggest that the mPFC receives information from the pulvinar to discriminate snake postures.

## Locations of The mPFC Neurons

**Figure 5:**
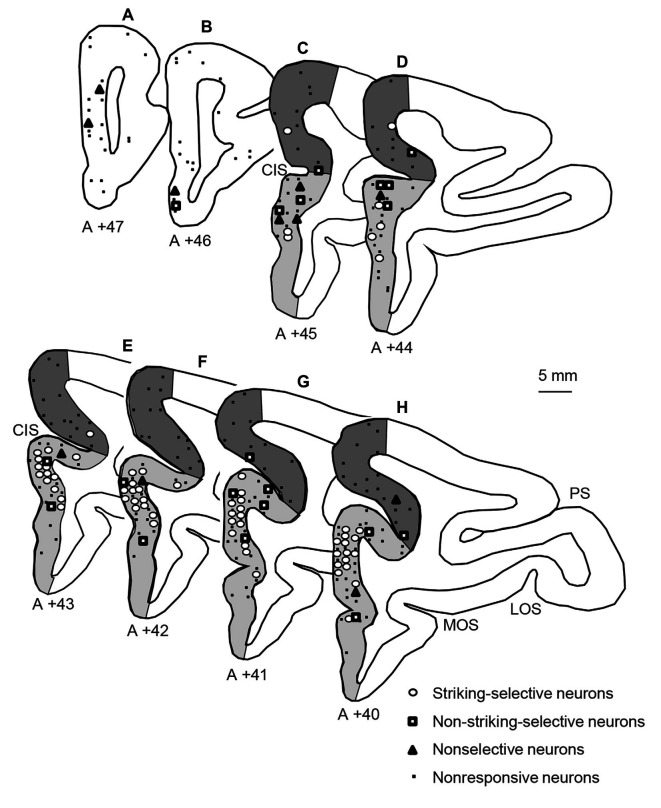
Locations of the three types of snake-responsive neurons in the mPFC. **(A–H)** Locations of mPFC neurons plotted in coronal sections at different A-P levels. The number under each section, distance (mm) anteriorly from the interaural line. Open circles, striking-selective neurons (*n* = 64); open squares, non-striking-selective neurons (*n* = 20); open triangles, nonselective neurons (*n* = 11); dots, nonresponsive neurons (*n* = 140). The mPFC was divided into three subareas: an anterior part of the mPFC (white areas in **A,B**), a dorsal part of the mPFC (dark gray areas in **C–H**), and a ventral part of the mPFC (light gray areas in **C–H**). PS, principal sulcus; LOS, lateral orbital sulcus; MOS, medial orbital sulcus; CIS, cingulate sulcus.

The histological procedures to identify recording sites in the mPFC were reported previously (Dinh et al., 2018). Briefly, after the last recording session, the monkeys were perfused under deep anesthesia, and brain sections were processed with Cresyl violet. Then, recording sites of mPFC neurons were stereotaxically determined based on the stereotaxic brain atlas of monkeys (*Macaca fuscata*; Kusama and Mabuchi, 1970). The mPFC was divided into three subareas (Dinh et al., 2018); the area anterior to the cingulate sulcus (anterior mPFC), area dorsal to the fundus of the cingulate sulcus (dorsal mPFC), and area ventral to the fundus of the cingulate sulcus (ventral mPFC; Figure 5). Consistent with the previous study in the mPFC (Dinh et al., 2018), the snake-responsive neurons were located mainly in the ventral mPFC (Figure 5). Especially, the striking-selective neurons were located in the ventral mPFC corresponding to the pregenual part of the ACC (i.e., rACC).

## Discussion

In the present study, we showed that the monkey mPFC neurons responded stronger and faster to snakes with striking postures compared with snakes with non-striking postures. Furthermore, population activity patterns of the mPFC neurons discriminated against snakes with striking postures from those with non-striking postures in epochs 2 and 3. Ethological studies reported that the striking speed of snakes is fast enough to preclude motor escape responses from mammal species (Penning et al., 2016). Given that biting strikes are generally preceded by striking postures (Arnold and Bennett, 1984; Greene, 1988), fast discrimination of snake posture may be critical to surviving agonistic encounters with snakes. Consistently, monkeys respond strongly to snake models with striking postures (Etting et al., 2014). We previously reported that mPFC and pulvinar neurons responded stronger and quicker to snakes compared with other animals (Le et al., 2013; Dinh et al., 2018), and that pulvinar neurons also discriminated against snakes with striking postures from those with non-striking postures (Le et al., 2014). Furthermore, MDS locations of snakes with and without striking postures in the mPFC were significantly correlated with those in the pulvinar, suggesting that the mPFC receives inputs directly from the pulvinar or indirectly *via* the amygdala since the pulvinar also sends projections to the amygdala (Rafal et al., 2015; Diano et al., 2017; Elorette et al., 2018), and the pulvinar and amygdala send projections to the mPFC (Porrino et al., 1981; Romanski et al., 1997). These results suggest that the mPFC and subcortical visual pathways are particularly sensitive to evolutionary threats (striking postures of snakes), which is consistent with the Snake Detection Theory, in which predations of primates by snakes are important selective forces to shape the primate brain to be sensitive to snakes (Isbell, 2006, 2009). These findings support the idea of the evolutionary origin of specific phobia, especially ophidiophobia (see “Introduction” section).

The striking-selective neurons were located mostly in the mPFC area roughly corresponding to the pregenual part of the ACC. Human imaging studies reported that phobogenic stimuli elicited activation in the rACC that roughly corresponds to the pregenual part of the ACC (see “Introduction” section), and that functional connectivity between the rACC and amygdala increased (Gold et al., 2015) and activity in the rACC covaried with activity in the amygdala (Carlson et al., 2009) in presence of threat stimuli. Previous electrophysiological studies reported that rACC neurons showed excitatory responses to aversive cues and inhibitory responses to rewarding cues (Takenouchi et al., 1999; Amemori and Graybiel, 2012) and that microstimulation of the rACC increased bias for avoidance behavior (Amemori and Graybiel, 2012), suggesting that activity in the rACC may be anxiogenic. Consistently, a meta-analysis of imaging studies of patients and at-risk groups suggests that the pregenual ACC is associated with the expression of emotional psychopathology (Marusak et al., 2016). Furthermore, cortical thickness in the pregenual ACC was increased in patients with animal phobia including ophidiophobia compared with healthy controls (Rauch et al., 2004). These findings suggest that the rACC, along with the amygdala, is an important brain region for the development of ophidiophbia.

Attentional bias to specific animals promotes anxiety and phobia (see “Introduction” section). The mPFC is reported to be involved in attentional allocation to various salient visual stimuli (Dalley et al., 2004; Guillem et al., 2011; Kaping et al., 2011; Mao et al., 2017). These findings suggest that neurons in the rACC, which receive inputs from the amygdala and pulvinar, may guide attention to focus on snakes. Consistent with this idea, gray matter volume in the rACC was positively correlated with covert attention to threatening stimuli (Carlson et al., 2012). The increases in attention bias to snakes might be associated with the pathogenesis of ophidiophobia. Human behavioral studies reported that training anxious subjects to guide attention to non-threatening faces from threatening faces reduced anxiety (Heeren et al., 2013). The same method may be applied to patients to treat ophidiophobia. On the other hand, the pregenual ACC (rACC) has bidirectional (reciprocal) connections with the amygdala (Calderazzo et al., 2020), and these bidirectional connections are excitatory (Laviolette et al., 2005; Bissière et al., 2008). It is reported that activity in the rACC is required to develop aversive conditioning in the amygdala (Laviolette et al., 2005; Bissière et al., 2008). These findings suggest that rACC neurons responsive to snakes may guide aversive conditioning in the amygdala. A human behavioral study using aversive conditioning reported that the association between snakes and unconditioned shock was more strongly formed than the association between other control objects and shock (Öhman and Mineka, 2003). These findings suggest that the rACC might initially and preferentially respond to snakes through direct inputs from the pulvinar. The rACC is also likely to facilitate amygdala-dependent conditioning processes, which in turn might further facilitate rACC responses to snakes. The increased activity in the rACC may promote attention to snakes, which may increase the likelihood of developing ophidiophobia. Taken together, these findings highlight the role of the rACC-amygdala circuits in the pathogenesis of specific phobia.

## Limitation

First, the responses to snakes could depend on low-grade visual features of the snake stimuli, but not on their postures. However, our previous study reported that the responses to snakes in the mPFC were decreased by scrambling of the stimuli using two different methods (quadrate-scrambling, in which snake images were cut into small fragments, and the fragments were randomly reassembled, and Fourier-scrambling, in which shapes were degraded while the global low-level visual features of the original snake images were maintained) (Dinh et al., 2018). These findings suggest that mPFC neurons responded to snake shapes (i.e., snake postures), but not to low-grade visual features of snake images.

Second, the mPFC neurons were recorded from the two monkeys. Since the proportions of snake-responsive and striking-selective neurons among the mPFC neurons were similar between the two monkeys (Supplementary Table 1), the data from the two monkeys were combined for further analyses. Although this way of analysis (analyses of combined data of neuronal responses recorded from a few animals) is relatively common in monkey neurophysiological studies (e.g., Tsao et al., 2006; Tsujimoto et al., 2010; Cavanaugh et al., 2020), further studies using more animals are required to test the present hypothesis.

## Data Availability Statement

The raw data supporting the conclusions of this article will be made available by the authors, without undue reservation.

## Ethics Statement

The animal study was reviewed and approved by the Ethical Committee for Animal Experiments in University of Toyama.

## Author Contributions

HNishij conceived the study and designed the experiment. HD and QL performed the experiment. QL and HNishij analyzed the data and wrote the article. HNishij, HNishim, JM, TS, RM, CT, and TO revised the article. All authors contributed to the article and approved the submitted version.

## Conflict of Interest

The authors declare that the research was conducted in the absence of any commercial or financial relationships that could be construed as a potential conflict of interest. The handling editor declared a past co-authorship with one of the authors RM.
