# Supplementary material for: Preferential Neuronal Responses to Snakes in the Monkey Medial Prefrontal Cortex Support an Evolutionary Origin for Ophidiophobia
Source: Front Behav Neurosci. 2021 Mar 24;15:653250. doi: 10.3389/fnbeh.2021.653250 (PMC8024491; doi:10.3389/fnbeh.2021.653250)
Supplement: Supplementary file 1 [file Data_Sheet_1.PDF]

Preferential neuronal responses to snakes in the monkey medial prefrontal cortex support an evolutionary origin for ophidiophobia

Supplementary information

Supplementary Table 1. Numbers of mPFC neurons, snake-responsive neurons, and striking-selective neurons recorded from individual monkeys.

	Monkey1	Monkey2	Total
No. of mPFC neurons	136	99	235
No. of snake-responsive neurons	51	44	95
No. of striking-selective neurons	36	28	64

The proportions of snake-responsive neurons among the mPFC neurons were not significantly different between the two monkeys [χ^2-test; $\chi^2(1) = 1.147$, $p = 0.284$]. Furthermore, the proportions of striking-selective neurons among the mPFC neurons were not significantly between the two monkeys [χ^2-test; $\chi^2(1) = 0.095$, $p = 0.758$].
